# Don’t touch: Developmental trajectories of toddlers’ behavioral regulation related to older siblings’ behaviors and parental discipline

**DOI:** 10.1111/sode.12440

**Published:** 2020-02-10

**Authors:** Sheila R. van Berkel, Ju‐Hyun Song, Richard Gonzalez, Sheryl L. Olson, Brenda L. Volling

**Affiliations:** ^1^ Forensic Family Science and Youth Care Studies Leiden University Leiden the Netherlands; ^2^ Department of Child Development California State University Dominguez Hills Carson CA USA; ^3^ Department of Psychology University of Michigan Ann Arbor MI USA

**Keywords:** behavioral regulation, developmental trajectories, early development, modeling, siblings, verbal and physical control

## Abstract

Behavioral regulation is one of the key developmental skills children acquire during early childhood. Previous research has focused primarily on the role of parents as socializing agents in this process, yet it is likely that older siblings also are influential given the numerous daily interactions between siblings. This exploratory longitudinal study investigated developmental heterogeneity in behavioral regulation during toddlerhood and the early preschool years (18 to 36 months) and relations with older siblings’ control and behavioral regulation while taking into account parental discipline. Toddlers were visited at home at 18, 24, and 36 months and observed during a gift‐delay task with their older sibling in 93 families. Behavioral regulation of both siblings and gentle and harsh control of the older sibling were coded during the sibling gift‐delay task, which was validated using parent‐reports of toddlers’ internalized conduct. Analyses revealed five distinct developmental trajectories among toddlers’ behavioral regulation, revealing different patterns of developmental multifinality and equifinality. Older siblings’ harsh control and parental discipline differed across toddler trajectory groups. Older siblings’ behaviors covaried with the toddlers’ behavioral regulation suggesting that older siblings may be acting as models for younger siblings, as well as disciplining and teaching toddlers to resist temptation.

## 1 INTRODUCTION

Early behavioral regulation is associated with a host of positive adjustment outcomes including higher academic performance and better social‐emotional functioning and psychological health in childhood and adulthood (Mischel et al., 2010; Moffitt, Poulton, & Caspi, 2013). Behavioral regulation—the ability to control one's behaviors in tempting situations and to inhibit prohibited behavior—develops dramatically in the early years (see Bridgett, Burt, Edwards, & Deater‐Deckard, 2015 for a review; Kochanska, Tjebkes, & Fortnan, 1998; Putnam, Spritz, & Stifter, 2002). During toddlerhood and the early preschool years, children rely predominantly on external guidance mostly from parents to regulate behavior (Kochanska & Aksan, 2006; Putnam et al., 2002), but siblings—especially older siblings with better regulatory abilities—also may play important roles in scaffolding the development of children's behavioral regulation. Similar to parents, older siblings control or monitor a younger sibling's misbehaviors (Van Berkel et al., 2017), and in turn, may contribute to the younger sibling's development of regulatory skills. Given the large individual differences in sibling dynamics and the developmental timing of regulatory skills, there is no doubt heterogeneity in the developmental patterns of behavioral regulation in early childhood. The primary goal of this exploratory investigation was to identify different trajectories of toddlers’ behavioral regulation from 18 to 36 months in a novel sibling gift‐delay task, and investigate whether these trajectories were related to older siblings’ behaviors and parental discipline. We refer to the younger siblings as toddlers for the remainder of this paper.

### 1.1 Development of behavioral regulation in the early years

Toddlerhood and the preschool years are important periods for the development of behavioral regulation and rule‐based compliance. The development of behavioral regulatory capacities begins around 8 to 10 months when infants use “spontaneous restraint” (e.g., do not touch a plant placed nearby; see Kochanska et al., 1998), and continues into the second year, when children are capable of inhibiting prohibited behaviors upon parental requests (Kochanska et al., 1998). Even with the advent of behavioral self‐regulation, children remain dependent on external guidance to regulate their behaviors during toddlerhood. The timing of when young children are able to inhibit their behavior in delayed‐gratification (“don't touch”) paradigms varies across individuals (e.g., Kochanska, Coy, & Murray, 2001), but is moderately stable from the preschool years onward (Bridgett et al., 2015). Because of the significance of early behavioral regulation for predicting children's problem behaviors and school readiness (e.g., Blair & Raver, 2015; Eisenberg et al., 2000), understanding individual differences in behavioral regulation trajectories across the early years is important for developmental research.

The first aim of the current study was to examine toddlers’ behavioral regulation trajectories between 18, 24, and 36 months based on their ability to inhibit touching in a modified gift‐delay task involving their older siblings. Although most young children eventually learn to inhibit their responses, the pathways by which this is accomplished may differ (Dong, Wang, Lu, Liang, & Xing, 2018; Friedman, Miyake, Robinson, & Hewitt, 2011; Kochanska et al., 2001). The development of self‐regulation may not always follow a linear pattern, and, as a result, the ability to inhibit behavior may actually be a developmental process during which children are able to inhibit behavior in some situations, but not others, resulting in inconsistent responding across time before performance is consolidated into a stable pattern. For instance, some children may be able to inhibit their response at an early age (24 months), whereas others may not manage this developmental task until later (36 months; Friedman et al., 2011).

### 1.2 The role of older siblings in toddler regulation

Although most studies consider the role of parents in the development of behavioral regulation, older siblings also provide external regulation for toddler siblings in the form of both verbal and physical control (Van Berkel et al., 2017). Because older siblings are cognitively and socio‐emotionally more mature than their toddler siblings, they often have more advanced behavioral regulation and a better understanding of the consequences of transgressions (Kochanska & Aksan, 2006; Vaish, Missana, & Tomasello, 2011). Furthermore, older siblings generally take the lead in sibling interactions and often function as role models for toddlers (Dunn, 1983; Howe, Ross, & Recchia, 2011). Toddler siblings correspondingly are more inclined to imitate behaviors of older siblings spontaneously compared to their parents or peers (Howe, Rosciszewska, & Persram, 2018). Thus, older siblings may play a unique role in the development of toddlers’ behavioral regulation in addition to the influence of their parents.

As the second aim, we examined the relations between trajectories of toddlers’ behavioral regulation and their older siblings’ control and behavioral regulation observed during the gift‐delay paradigm. Whether older siblings have a positive influence on toddlers’ behavioral regulation may depend on their own behavioral regulation and the guidance and control they use during the task. In challenging situations where children are asked to inhibit their behavior (e.g., a delay of gratification task), older siblings may help toddlers in regulating behavior by explaining or reiterating the rules of conduct or using gentle control that encourages the toddlers’ willingness to cooperate, similar to research on parental control (Hastings, Utendale, & Sullivan, 2007; Kochanska & Aksan, 2006; Van IJzendoorn, 1997). Harsh control, in contrast, may hamper the development of intrinsically driven behavioral regulation of toddler siblings (Cecil, Barker, Jaffee, & Viding, 2012) because sibling aggression and forceful restraint may emotionally over‐arouse a toddler (Hoffman, 2000). This over‐arousal eventually may interfere with toddlers’ regulatory abilities and contribute to externalizing behavior (Tucker, Finkelhor, Turner, & Shattuck, 2013), which is supported by work showing that older siblings’ use of physically controlling teaching strategies with preschoolers was associated negatively with preschool siblings’ abilities to complete the task (Howe, Recchia, Della Porta, & Funamoto, 2012).

Siblings’ roles in toddlers’ development of regulation need to be viewed systemically, as siblings are imbedded within the family system (Feinberg, Solmeyer, & McHale, 2012). Toddler behavioral regulation can be affected both by parental discipline directed toward themselves, as well as toward their sibling (Blandon & Volling, 2008; Van Berkel et al., 2015; Volling, Blandon, & Gorvine, 2006). In line with social learning theory and ideas about social modeling of one's parents (Bandura, 1977), older siblings’ control may be similar to the discipline strategies used by their parents, which also may be related to toddlers’ behavioral regulation. As a final aim, therefore, we examined whether parental discipline directed to either sibling was related to behavioral regulation of either child, and to older siblings’ control. We also tested whether the significant effects of older siblings’ control across the trajectories of toddler behavioral regulation remained once we controlled for parental discipline in an effort to address the uniqueness of sibling effects.

### 1.3 The current study

The current study used a longitudinal approach with repeated measures to examine toddlers’ developmental trajectories of behavioral regulation (i.e., touching in a gift‐delay task with siblings) from 18 to 36 months of age, and how these trajectories differed with respect to older siblings’ touching and control strategies observed during the task. Because no prior research has observed toddlers in a delay of gratification task with their siblings, the analyses were by necessity exploratory and descriptive in nature. There were three specific aims to the current study: (a) to identify different developmental trajectories of toddlers’ behavioral regulation (touching) across 18, 24, and 36 months of age; (b) to determine whether the older siblings’ touching and behavioral control differed across trajectories of toddler behavioral regulation; and (c) to investigate whether any differences in older siblings’ behavior across trajectories changed or remained the same after controlling for parental discipline directed to either sibling.

#### 1.3.1 Hypothesized trajectory patterns

Based on the extant literature on early self‐regulation (Bridgett et al., 2015; Dong et al., 2018; Friedman et al., 2011; Kochanska et al., 2001), we expected several potential trajectory patterns for toddler behavioral regulation across 18 to 36 months. First, we expected most children would show a pattern of stable improvement (linear decline in touching over time) in their behavioral regulation from 18 to 36 months. A second potential pattern would reflect an increase in touching from 18 to 24 months, followed by a decline from 24 to 36 months, which would be consistent with the documented increases in autonomy seeking, assertiveness, and oppositional behavior that emerge during the second year (Crockenberg & Litman, 1990; Forman, 2007; Kuczynski & Kochanska, 1990); a period sometimes referred to as the “terrible twos.” Another possible pattern would involve stable patterns of behavioral regulation over time evincing no change, such as the inability to delay touching at any time (stable high) or little touching across time (stable low). A final pattern might reveal a steady linear increase in touching (noncompliance) from 18 to 36 months similar to chronic patterns noted in the development of externalizing behavior from 2 to 5 years of age (Hill, Degnan, Calkins, & Keane, 2006). Because most children improve in behavioral regulation across this period (Dong et al., 2018; Friedman et al., 2011), we expected few toddlers to show this last pattern, but should such a group emerge, they would be worth investigating further given the problematic nature of increased noncompliance. Because this is the first study to examine different trajectories of children's early behavioral regulation in a sibling gift‐delay task, an exploratory descriptive approach was used in which we created a priori groups of toddlers in line with these hypothesized trajectories based on the frequency of toddler touching at each of the three times (described in greater detail later).

#### 1.3.2 Planned developmental comparisons

To examine differences in parent and sibling behaviors between resulting trajectory patterns, we planned a series of a priori comparisons based on notions of *multifinality*—a process by which similar beginnings take different developmental paths—and *equifinality*—a process by which different trajectories result in similar developmental outcomes (Cicchetti & Rogosch, 1996). This approach allowed us to examine whether older siblings’ behaviors differed and changed over time similar to changes observed in toddler touching, which would be consistent with a *sibling modeling* hypothesis (i.e., toddlers imitate their older siblings so both toddler and older sibling touching would be related over time and show similar trajectory patterns). In line with a *sibling socialization* hypothesis, older siblings’ use of harsh or gentle control might also differ across toddlers’ touching trajectories; however, we advanced no specific a priori hypotheses as to how older siblings’ control would change over time in the specific trajectory patterns given the exploratory nature of this research and the fact that no prior study has examined relations between sibling and toddler regulation in the manner described here. In a similar vein, we investigated whether parental punitive discipline (by mothers and fathers) differed across toddler trajectories given the strong links between parental control strategies and children's behavioral regulation in other delay of gratification tasks (Kochanska, Brock, & Boldt, 2016; Song, Miller, Leung, Lumeng, & Rosenblum, 2018). Here, we expected parents to use more control during times when toddlers evinced more touching (i.e., noncompliance). Finally, we investigated whether any significant differences in older siblings’ behaviors remained once we controlled for parental discipline. Given the significant and unique effects of sibling socialization in early childhood (Fagan & Najman, 2003; Ostrov, Crick, & Stauffacher, 2006; Pike & Oliver, 2017), we expected differences in older siblings’ behaviors to remain significant after controlling for parental discipline.

## 2 METHOD

### 2.1 Participants

The sample consisted of two‐parent, two child families participating in a longitudinal study examining relations between family functioning and toddler self‐regulation. The study was conducted in two phases. Phase I involved the initial recruitment of 241 families living in the Midwestern U.S, during the last trimester of mother's pregnancy with the second child, and then following them at 1, 4, 8, 12 months after the birth (see Volling et al., 2017, for recruitment details). Phase II involved follow‐up assessments when the second‐born children were 18, 24, and 36 months of age. Data for the current report are from Phase II in which toddler self‐regulation and sibling relationships were the focus. The total number of participating families varied across the three time points with 155 participating at 18 months, 140 at 24 months, and 133 at 36 months.

A total of 93 families completed the modified sibling gift‐delay task at 18, 24, and 36 months. Demographics (family income, parents’ age, older siblings’ age, parental education, years of marriage, and race) of the 93 families with complete data at all three times were not different from those of the total 155 families that participated at 18 months, *p*s >.07. Little's (1988) MCAR test showed that data were missing completely at random, *χ^2^* (315) = 335.50, *p* = .21. The distribution of sibling gender configuration (older‐younger) included 19 boy‐boy (20%), 22 girl‐girl (24%), 16 boy‐girl (17%), and 36 girl‐boy (39%). At the 18‐month time point, firstborn children were between 30 and 85 months old (*M* = 49.4, *SD* = 10.4), mothers were between 25 and 43 years of age (*M* = 33.6, *SD* = 3.7), and fathers were between 27 and 48 years of age (*M* = 34.6, *SD* = 4.0). Most participating parents self‐identified as European American (86.5% of both mothers and fathers). Of the mothers, 5.2% were African American, 3.2% were Asian American, 3.2% were Hispanic, and 1.9% reported “other” ethnicity; 4.5% of fathers were African‐American, 3.9% were Asian American, 3.2% were Hispanic, and 1.9% reported “other” ethnicity. Annual income ranged from less than $20,000 to more than $100,000, with the median of $60,000–$99,999. With regard to educational level, most of the mothers (87.1%) and fathers (79.4%) had at least a bachelor's degree.

### 2.2 Procedure

At 18, 24, and 36 months, families were invited to participate in both a home and a laboratory observational session. As part of the home visits, both siblings were presented with a wrapped gift and asked not to touch it while the experimenter left the room for three minutes, leaving both siblings together. Information for the current study was obtained from behavioral observations during this ‘*sibling gift‐delay task*’, which were video recorded for later coding of children's behavioral regulation (i.e., touching gift), affect (e.g., positive affect), sibling interactions (e.g., sibling control, compliance), and coping behaviors (e.g., self‐soothing, comfort seeking). The focus here was on touching the gift as a form of noncompliance and hence, poorer behavioral regulation, and the older siblings’ control strategies.

### 2.3 Measures

#### 2.3.1 Sibling gift‐delay paradigm

At each time‐point, each sibling's behavioral regulation (i.e., touching) was observed during the 3‐min gift‐delay paradigm. At 18 and 24 months, each child was presented with a small gift, wrapped, but with no bow. An experimenter pretended she forgot the bows and asked that the children not touch the gifts until she returned and then left the room for three minutes. At 36 months, the task was slightly different in that the experimenter pretended she forgot the nametags for the presents, instead of the bows. As such, children did not know which gift was theirs once the experimenter left. Both parents were present in the room completing questionnaires and were instructed not to enforce the “don't touch” rule, but to intervene in sibling conflict if they judged it necessary to do so. For each 15‐s interval (12 total), trained coders rated whether the children *touched* (coded 1) or *did not touch* (coded 0) one of the two gifts. A total score for each sibling was created by summing the codes across the 12 intervals for the two gifts for a possible score of 0 (touched neither gift in any of the 12 intervals) to 24 (touched both gifts in all intervals). Different coders rated each sibling within the same family at each time point to guarantee independence among coders. Inter‐rater reliability (Cohen's kappa using approximately 15% of cases) ranged from *κ* = 0.83–0.91 (*M* = 0.88). We also conducted *post hoc* analyses separately by whether one's own gift or one's sibling's gift was touched (see results section).

#### 2.3.2 Validity of the sibling gift‐delay

Because the gift‐delay task was modified to include both siblings, concurrent correlations were computed between children's touching and parental reports of internalized conduct obtained from the *My Child Questionnaire* (MCQ; Kochanska, DeVet, Goldman, Murray, & Putnam, 1994) completed by mothers and fathers at 18, 24, and 36 months. This scale assessed children's abilities to comply autonomously with rules when not monitored, which is consistent with the expectations of the gift‐delay task. Internal consistency for both mothers’ and fathers’ reports across the three time points was above 0.80, and because mothers’ and fathers’ reports were significantly correlated at each time for both older, *r* = .54–.64, all *p*s < .01, and toddler siblings, *r* = .38–.61, all *p*s < .01, parent reports were averaged to create more robust composites. As can be seen in Table 1, the frequency of toddlers’ touches at 18 months was negatively related to their internalized conduct at 18 months, and toddlers’ touching at 24 and 36 months was associated negatively with their internalized conduct at 36 months. There were no relations between older siblings’ touching and parent reports of their internalized conduct at any time. Thus, the sibling gift‐delay task appears to provide a valid assessment of the toddlers’ (the focal children of this study) behavioral regulation across this period of development.

**Table 1:** Descriptive statistics and correlations between touching, internalized conduct, older siblings’ control, and parental discipline

	1	2	3	4	5	6	7	8	9	10	11	12	13	14	15	16	17	18	19	20	21	22	23	24
Time 1−18 m																								
1.YS T																								
2.YS IC	**−0.21** *																							
3.OS T	**0.26** *	**−0.17**																						
4.OS IC	**−0.19**	**0.41** **	**−0.05**																					
5.OS HC	**0.13**	**−0.23** *	**0.34** **	**−0.14**																				
6.OS GC	**0.09**	**−0.04**	**0.08**	**0.01**	**0.11**																			
7.PPD YS	**0.27** **	**−0.31** **	**0.18**	**−0.04**	**0.13**	**−0.06**																		
8.PPD OS	**0.18**	**−0.11**	**0.08**	**−0.27** **	**0.10**	**−0.07**	**0.61** **																	
Time 2−24 m																								
9.YS T	0.14	−0.11	0.19	−0.02	−0.07	−0.10	0.12	0.01																
10.YS IC	−0.15	0.68**	−0.07	0.27**	−0.06	−0.09	−0.20	−0.03	**−0.08**															
11.OS T	0.02	−0.18	0.25*	−0.09	0.16	0.11	0.04	0.01	**0.54** **	**−0.13**														
12.OS IC	−0.13	0.38**	−0.10	0.79**	−0.14	−0.11	0.02	−0.22**	**−0.05**	**0.33** **	**−0.20**													
13.OS HC	0.12	−0.10	0.14	−0.13	−0.07	−0.11	0.04	0.04	**0.38** **	**−0.14**	**0.49** **	**−0.20**												
14.OS GC	0.03	−0.07	0.01	−0.08	0.24*	−0.02	0.07	0.03	**0.25** *	**−0.09**	**0.04**	**−0.01**	**−0.01**											
15.PPD YS	0.08	−0.20	0.18	−0.08	0.15	0.01	0.71**	0.58**	**0.20**	**−0.16**	**0.11**	**−0.07**	**0.13**	**0.10**										
16.PPD OS	0.10	−0.10	−0.05	−0.23	0.09	0.00	0.53**	0.74**	**0.13**	**−0.08**	**0.10**	**−0.22** *	**0.19**	**−0.03**	**0.71** **									
Time 3−36 m																								
17.YS T	−0.12	−0.05	0.03	−0.02	0.12	0.12	−0.16	−0.21*	0.18	−0.17	0.25*	−0.09	0.12	0.06	−0.07	−0.20								
18.YS IC	−0.03	0.50**	−0.14	0.32**	−0.09	−0.13	−0.22*	−0.08	−0.27*	0.67**	−0.22*	0.31**	−0.20	−0.08	−0.24*	−0.15	**−0.35** **							
19.OS T	−0.15	−0.09	0.02	−0.02	0.14	0.06	−0.12	−0.12	0.04	−0.10	0.03	0.00	−0.03	−0.08	0.01	−0.10	**0.51** **	**−0.15**						
20.OS IC	−0.20	0.36**	−0.19	0.74**	−0.13	−0.12	0.02	−0.18	−0.04	0.18	−0.12	0.79**	−0.17	0.02	−0.03	−0.13	**0.10**	**0.25** *	**0.08**					
21.OS HC	−0.21*	−0.16	−0.01	0.00	0.02	0.07	−0.13	−0.11	0.16	−0.08	0.36**	−0.01	0.19	0.11	−0.00	−0.12	**0.37** **	**−0.12**	**0.28** **	**−0.10**				
22.OS GC	−0.04	−0.10	−0.00	0.22*	0.49**	0.03	−0.08	−0.00	−0.00	−0.08	0.24*	−0.23*	0.11	0.05	−0.01	−0.04	**0.39** **	**−0.24** *	**0.24** *	**−0.15**	**0.13**			
23.PPD YS	0.09	−0.19	0.05	−0.05	−0.01	−0.02	0.63**	0.45**	0.14	−0.22*	−0.08	−0.00	0.06	0.04	0.67**	0.50**	**−0.04**	**−0.35** **	**0.00**	**−0.00**	**−0.04**	**−0.05**		
24.PPD OS	0.22*	−0.28*	0.06	−0.25*	0.04	0.14	0.42**	0.50**	0.10	−0.19	0.02	−0.19	0.10	−0.01	0.44**	0.61**	**−0.10**	**−0.13**	**0.03**	**−0.25** *	**−0.07**	**−0.08**	**0.60** **	
*Mean*	6.52	3.25	7.92	3.97	3.43	1.62	4.18	4.73	5.25	3.35	4.20	4.08	2.67	1.58	4.54	4.66	2.03	3.56	1.42	4.20	0.53	0.97	4.87	4.63
*SD*	4.84	0.57	6.32	0.60	3.16	1.61	0.82	0.81	4.64	0.64	4.63	0.60	3.46	2.22	0.94	0.97	2.89	0.60	2.17	0.73	1.27	1.61	1.04	0.89

Note

Bolded area represents correlations within a time point.

Abbreviations: GC, gentle control; HC, harsh control; IC, internalized conduct; OS, older sibling; PPD, parental punitive discipline; T, amount of touching; YS, younger sibling.

* *p* < .05;

** *p* < .01.

#### 2.3.3 Older siblings’ control

Older **s**iblings’ control strategies also were observed during the gift‐delay tasks. For each 15‐s interval, harsh and gentle control (verbal and physical) attempts to control toddlers’ touching were rated *present* (coded 1) or *absent* (coded 0) and included (a) *harsh verbal control—*raising their voice, posing a threat, or delivering a harsh command; (b) *harsh physical control—*forcefully touching the toddler's hand in order to remove it from the gift or restraining the toddler, blocking or grabbing the gifts, or forcefully interfering with the toddler when touching the gift (e.g., pulling the toddler away, moving the gifts away); (c) g*entle verbal control*‐ a polite request or gentle command (e.g., using the word please, or a calm and gentle voice with positive or neutral affect), explaining the rules, simply repeating the researcher's request, and reassuring or distracting the toddler; and (d) g*entle physical control—*gently touching the toddler's hand to remove it from the prohibited gift, gently removing the gift, or creating a barrier between the gift and the toddler. Scores across the 12 intervals were summed for a total score ranging from 0 to 12. The mean Kappas across three time points were *κ* = 0.74 for *gentle* verbal control, *κ* = 0.63 for *gentle* physical control *κ* = 0.75 for *harsh* verbal control, and *κ* = 0.87 for *harsh* physical control. Because verbal and physical forms of gentle, *r* = .18–.46, all *p*s < .09, and harsh, *r* = .35–.62, all *p*s < .01, control were correlated at each of the three time points, we summed each into a gentle control and a harsh control composite for the older siblings at each of the three times.

#### 2.3.4 Parental discipline

To obtain a measure of parental punitive discipline, mothers and fathers completed 10 items that included verbal reprimands (tell child “don't do that”), harsh verbal discipline (shame/embarrass child), and harsh physical discipline (grab, shake or restrain child) adapted from Olson and Sameroff (1997). At 18, 24, and 36 months, parents indicated how often they used, in an average week, a particular discipline strategy for each sibling on a scale ranging from 1 = *not at all* to 5 = *several times a day*. Internal consistency for both mothers’ and fathers’ reports across the three time points was above 0.70. Because the mothers’ and fathers’ reports were significantly correlated at each time for discipline used with both the older, *r* = .41–.46, all *p*s < .01, and toddler siblings, *r* = .42–.53, all *p*s < .01, mothers’ and fathers’ reports were averaged to create more robust composites of punitive discipline used with each sibling. 

## 3 RESULTS

### 3.1 Preliminary analyses

Data inspection showed that older siblings’ touching, harsh control, and gentle control were skewed positively. Analyses with inverse transformations, however, yielded similar results; therefore, the analyses with non‐transformed variables are presented for ease of interpretation. Means, standard deviations and correlations among all variables are presented in Table 1. Touching and internalized conduct were positively related between siblings at each time point. Stability of internalized conduct over time was found for both siblings, whereas stability in touching behavior over time was only found between 18 and 24 months for older siblings. Older siblings’ touching was related positively to their use of harsh control at all measurement occasions, whereas older siblings’ gentle control was related only at 36 months to their touching. Moreover, older siblings’ harsh and gentle control strategies were related positively to toddlers’ touching at 24 and 36 months. Older siblings’ harsh control at 18 months and their gentle verbal control at 36 months were related negatively to toddlers’ internalized conduct concurrently. Older siblings’ harsh control at 18 months was related positively to gentle control at 24 and 36 months. Punitive parental discipline (PPD) was related positively between siblings at each time point as well as across time. For both the older sibling and the toddler, PDD was related negatively to internalized conduct at each time point except for toddlers at 24 months. Notably, none of the behaviors of the toddler or the older sibling (i.e., touching of both children and older siblings’ control) were associated with PPD at any time point, with the only exception being toddlers’ touching at 18 months.

Gender of the toddler and gender constellation of the sibling dyad were unrelated to toddlers’ touching behavior, and were not considered further in analyses. Gender of the older sibling was related to toddlers’ touching at 24 months, *t*(91) = −2.15, *p* <.05, indicating that toddlers with an older sister touched the gifts more often, *M* = 6.03, *SD* = 4.68, than toddlers with an older brother, *M* = 3.94, *SD* = 4.33. Although age of the older sibling was not related to toddlers’ touching behavior, *p*s > .25, or their own touching behavior, *p*s > .17, we decided because of the large age range of older siblings to include both gender and age of the older siblings as covariates in all analyses.

To examine the developmental pattern of touching for the entire sample, a 3 (time) × 2 (sibling) Repeated Measures (RM)‐ANCOVA with touching as the dependent variable and older sibling's gender and age as covariates, was conducted using the general linear model of SPSS 23.0. This revealed a significant main effect of time, *F*(2, 180) = 10.05, *p* < .001, $\eta_{p}^{2}$ = 0.10, and a significant interaction between time and sibling, *F*(1.7, 152.8) = 3.75, *p* < .05, $\eta_{p}^{2}$ = 0.04 (means and *SD*s are presented in Table 1). Because the assumption of sphericity was violated, Greenhouse‐Geisser corrections were used in this and all subsequent omnibus ANCOVA analyses. Bonferroni‐corrected *post‐hoc* tests revealed that at both 24 and 36 months, toddlers touched the gifts more often than their older siblings, but not at 18 months. Further, both toddlers and older siblings, on average, showed a significant decrease in touching across the three time points as indicated by a significant linear within‐subjects contrast of time, *F*(1, 90) = 18.07, *p* < .001, $\eta_{p}^{2}$ = 0.17, and a nonsignificant quadratic contrast, *p* = .89 (Table 1).

### 3.2 Developmental trajectories of toddlers’ behavioral regulation

**Figure 1:**
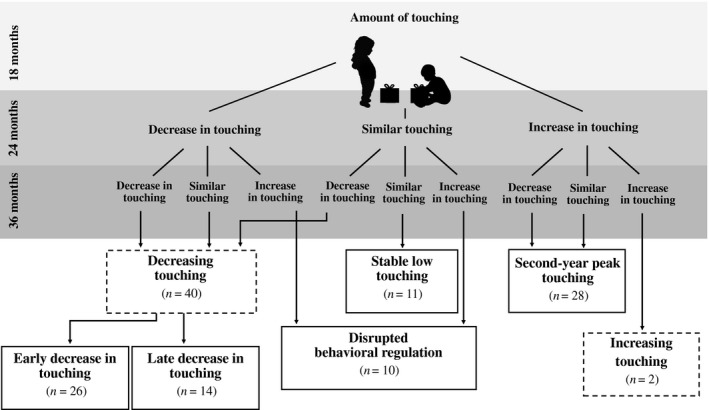
Developmental trajectory groups of children’s behavioral regulation from 18 to 36 months. Touching refers to the total number of times the target child touched the prohibited gifts during a 3‐minute sibling gift‐delay task

Toddlers were classified into different groups based on whether their frequency of touching behavior *decreased* (i.e., touched less often), *remained stable* (i.e. touched as often or one time more or less often as the previous observation) or *increased* (i.e., touched more) across 18 to 36 months (see Figure 1). Four different developmental trajectory groups were initially identified: (a) *decreasing touching* (*n* = 40, 43%) in which toddlers showed a pattern of decrease in touching from 18 to 36 months; (b) *stable low touching* (*n* = 11, 12%), in which toddlers showed little touching across 18 to 36 months; (c) *second*‐*year peak* (*n* = 28, 30%) in which toddlers touched the prohibited gifts during the task at 24 months more frequently than they did at 18 and 36 months; and (d) *disrupted behavioral regulation* in which toddlers touched the prohibited gifts during the task at 24 months less frequently or as frequently as they did at 18 months, but then rebounded and touched the gifts more frequently at 36 months (*n* = 12, 13%). As expected, only two toddlers (2%) showed an increase in touching from 18 to 36 months so they were dropped from further analyses.

**Figure 2:**
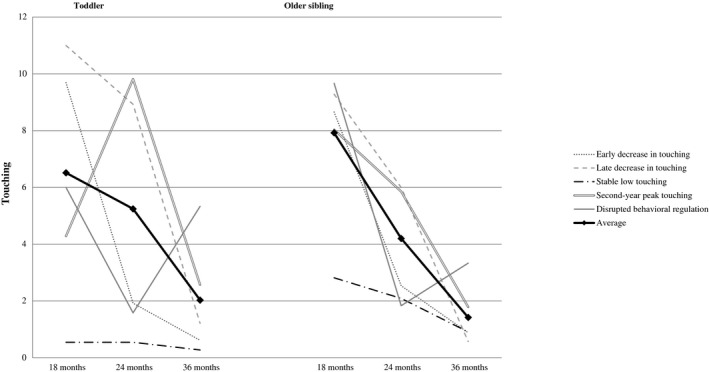
Patterns of touching over time of older siblings and toddlers for the different developmental trajectory groups of toddlers’ behavioral regulation The solid black line represents the average change of the entire sample toddlers and older siblings.

Spaghetti plots of the developmental trajectory groups were examined to confirm visually homogeneity in the patterns within each group. In doing so, two distinct developmental patterns were noted within the *decrease in touching* group, which were considered worthy of further follow‐up because of the timing of the decreases across the 18 months. One subgroup (*n = *26, 29%), labeled *early decrease in touching* (18–24 months), showed a greater decrease in touching from 18 to 24 months compared to 24 to 36 months, and the second subgroup (*n = *14, 15%), *late decrease in touching* (24–36 months)*,* showed a greater decrease in touching from 24 to 36 months than 18 to 24 months. Thus, there were five identifiable groups used for further analyses (see Figure 2). To confirm the labeling of the five resulting groups, a 3 (time) × 5 (group) RM‐ANCOVA with toddlers’ touching as the dependent variable and with older siblings’ age and gender as covariates was conducted. A significant interaction between time and trajectory group emerged, confirming the group differences (see Table 2). Both linear, *F*(4,84) = 21. 93, *p* < .001, and quadratic, *F*(4,84) = 68. 64, *p* < .001, within‐subjects contrasts were significant, substantiating the different trajectories over time. Bonferroni‐corrected *post‐hoc* tests confirmed significant differences across time and between groups except for the *stable low touching group* which showed no changes in touching over time. In the *disrupted behavioral regulation* group, the frequency of touching at 24 months differed from the other time points whereas frequency of touching at 18 and 36 months was similar.

**Table 2:** Differences in toddler touching, older sibling touching, their use of physical and verbal control, and parental discipline at 18, 24, and 36 months between toddler behavioral regulation trajectories

	18 months	24 months	36 months		Time		Time* Behavioral regulation trajectory	
	*M (SD)*	*M (SD)*	*M (SD)*		*F*	*η_p_^2^*	*F*	*η_p_^2^*
Toddlers touching					10.22**	0.11	35.84**	0.63
Early decrease	^1^9.69 (4.10)^a^	^1^1.92 (2.24)^b^	^1^0.62 (0.90)^c^	*df*	(1.7, 143.6)		(6.8, 143.6)	
Late decrease	^1^11.00 (3.03)^a^	^2^8.93 (2.25)^b^	^1^1.21 (1.58)^c^					
Stable low	^2,3^0.55 (0.93)	^1^0.55 (0.69)	^1^0.27 (0.47)					
Second‐year peak	^2,4^4.29 (2.95)^a^	^2^9.82 (3.21)^b^	^2,3^2.57 (3.47)^c^					
Disrupted	^2,4^6.00 (4.55)^a^	^1^1.58 (1.31)^b^	^2,4^5.33 (2.46)^a^					
Older siblings touching					6.04**	0.07	2.88**	0.12
Early decrease	^1^8.65 (6.07)^a^	^1^2.54 (4.02)^b^	^1^0.88 (1.56)^c^	*df*	(1.6, 138.4)		(6.6, 138.4)	
Late decrease	^1^9.29 (4.98)^a^	^2^6.00 (4.93)^a^	^1^0.57 (1.16)^b^					
Stable low	^2^2.82 (2.82)	^1^2.09 (3.73)	^1^0.91 (1.38)					
Second‐year peak	^1^8.04 (6.93)^a^	^2^5.86 (3.88)^a^	^1^1.79 (2.85)^b^					
Disrupted	^1^9.67 (7.73)^a^	^1^1.83 (2.72)^b^	^2^3.33 (2.06)^b^					
Older siblings harsh control					0.60	0.01	2.43*	0.10
Early decrease	^1^3.38 (3.24)^a^	2.00 (3.52)^a^	0.38 (0.85)^b^	*df*	(1.6, 130.7)		(6.2, 130.7)	
Late decrease	^3^4.42 (2.53)^a^	^1^3.93 (3.45)^a^	0.29 (0.61)^b^					
Stable low	^1,4^1.64 (2.25)	^2^0.82 (1.40)	0.45 (0.69)					
Second‐year peak	^1^2.64 (2.25)^a^	^1^3.64 (3.93)^a^	0.75 (1.76)^b^					
Disrupted	^2^5.50 (4.71)^a^	1.75 (2.05)^b^	0.25 (0.45)^b^					
Older siblings gentle control					0.82	0.01	1.17	0.05
Early decrease	1.81 (1.67)	1.23 (2.25)	0.81 (1.23)	*df*	(1.8, 152.3)		(7.3, 152.3)	
Late decrease	1.14 (1.10)	2.36 (2.21)	0.71 (1.38)					
Stable low	1.45 (2.11)	0.81 (0.87)	0.27 (0.65)					
Second‐year peak	1.64 (1.64)	1.89 (2.67)	0.93 (1.21)					
Disrupted	2.00 (1.65)	1.33 (1.78)	1.83 (2.69)					
Parental punitive discipline toddler					2.44	0.03	2.33*	0.10
Early decrease	4.30 (1.02)^a^	4.62 (0.95)^b^	4.81 (1.03)^b^	*df*	(1.4, 48.12)		(5.44, 48.12)	
Late decrease	4.54 (0.70)	4.69 (1.05)	4.74 (1.15)					
Stable low	3.86 (0.60)^a^	4.32 (0.98)^b^	4.57 (0.97)^b^					
Second‐year peak	4.09 (0.78)^a^	4.64 (0.88)^b^	5.02 (1.13)^c^					
Disrupted	4.15 (0.75)^a^	4.18 (1.13)^a^	5.16 (0.85)^b^					
Parental punitive discipline older sibling					0.47	0.01	1.45	0.07
Early decrease	4.91 (0.92)	4.76 (1.06)	4.76 (0.86)	*df*	(1.9, 49.14)		(3.5, 49.14)	
Late decrease	5.18 (0.60)	5.09 (0.69)	4.76 (1.04)					
Stable low	4.95 (1.01)	4.75 (1.19)	4.50 (1.48)					
Second‐year peak	4.57 (0.57)	4.73 (0.64)	4.65 (0.60)					
Disrupted	4.31 (0.67)	4.03 (1.16)	4.45 (0.60)					

Notes

Results control for older siblings’ gender and age. Superscripts numbers 1 and 2, and 3 and 4 note significant differences across groups within time for each variable. Superscripts letters a and b note significant differences across time within group

* *p <* .05;

** *p <* .01.

#### 3.2.1 Touching own or sibling gift

As a follow‐up, we examined whether trajectory groups differed based on touching one's own or the sibling's gift using scores at 18 and 24 months. For touching at 36 months this distinction between touching one's own or the sibling's gift could not be made because ownership was unknown to the children during the delay task (see Method). Two 2 (time) × 5 (group) × 2 (touching own gift vs. sibling gift) RM‐ANCOVAs were conducted (controlling for the older siblings’ age and gender). These analyses showed that toddlers and older siblings of all developmental trajectories at both 18 and 24 months, touched their own gift more often than their siblings’ gift, toddlers: *F* (1, 80) = 26.91, *p* < .001, $\eta_{p}^{2}$ = 0.25 *M*_own gift_ = 4.03, *SD*_own gift_ = 0.26, *M*_sibling gift_ = 1.44, *SD*_sibling gift_ = 0.15; older siblings: *F*(1, 80) = 5.61, *p* < .05, $\eta_{p}^{2}$ = 0.07, *M*_own gift_ = 3.57, *SD*_own gift_ = 0.32, *M*_sibling gift_ = 2.11, *SD*_sibling gift_ = 0.22.

### 3.3 Older siblings’ touching and control behavior

To investigate longitudinal variations in older siblings’ touching and use of control in relation to the toddlers’ developmental trajectories over time, a RM‐MANCOVA was conducted, with older siblings’ touching, and harsh and gentle control as dependent variables, time (3) as the repeated measure, trajectory groups (5) as the between‐subjects factor, and older siblings’ gender and age as covariates. Results revealed a multivariate effect of time, *F*(6, 334) = 2.19, *p* = .044, $\eta_{p}^{2}$ = 0.04, and a multivariate interaction effect of time by trajectory group, *F*(24, 504) = 2.08, *p* = .010, $\eta_{p}^{2}$ = 0.08. Follow‐up univariate RM‐ANCOVAs showed a significant main effect of time for older siblings’ touching, revealing a significant decrease over time (see Table 2), which was substantiated by a significant linear within‐subjects contrast, *F* (1,84) = 8.54, *p* < .01, $\eta_{p}^{2}$ = 0.09. Moreover, significant time by group interactions for older sibling's touching and harsh control were found (see Table 2 for all significant *post‐hoc* comparisons between groups and across time). The interaction for older siblings’ touching showed only a significant quadratic within‐subjects contrast, *F* (1,84) = 4.67, *p* < .01, $\eta_{p}^{2}$ = 0.18, whereas the interaction for older siblings’ harsh control only showed a significant linear contrast, *F* (1,84) = 3.92, *p* < .01, $\eta_{p}^{2}$ = 0.16. These contrasts indicated that older siblings in the different trajectory groups showed different touching trajectories over time, although there were no differences in the linear decrease in older siblings’ touching between groups. In addition, the linear contrast for harsh control indicated that the linear decrease in older siblings’ harsh control differed between trajectory groups. These interaction patterns, quadratic for older siblings’ touching and linear for harsh control, were confirmed by the results of the planned pairwise comparisons described below and presented in Table 2.

In line with our goal to compare trajectories reflecting differing developmental pathways, planned group comparisons were conducted using Bonferonni corrected tests to examine differences in older siblings’ touching and harsh control behaviors across time and groups to follow‐up the significant interactions for these variables noted above. These comparisons were designed to test for multifinality (one comparing groups with similar changes between 18 and 24 months and two comparing groups with different trajectories but starting the same at 18 months) and equifinality (one comparing groups increasing between 24 and 36 months and two comparing groups with different trajectories but ending at the same point at 36 months; also see Figure 2 for the different toddler trajectory patterns).

#### 3.3.1 Developmental multifinality

Comparisons between the *early decrease* and *late decrease* groups—both starting at similar levels of touching but having different paths over time resulting in less touching at 36 months—were conducted first and revealed differences in the older siblings’ touching over time consistent with the decline in toddler touching (see Table 2). Older siblings of toddlers in the *early decrease* group significantly declined in their touching from 18 to 24 months, and from 24 to 36 months, whereas for older siblings in the *late decrease* group, touching declined significantly only between 24 and 36 months, similar to toddlers. Older siblings in the *late decrease* group touched the gifts more often at 24 months compared to older siblings in the *early decrease* group (see Table 2). Changes in older siblings’ harsh control showed a similar pattern with both groups using more harsh control at 18 and 24 months, but decreasing in harsh control from 24 to 36 months; there were no differences between groups at any of the three time points.

The second comparison was conducted between the *early decrease* and *disrupted behavioral regulation* groups; toddlers in both groups showed declines in touching from 18 to 24 months, but the *early decrease* group continued to decline whereas the *disrupted regulation* group showed an increase in touching from 24 to 36 months. Older siblings of the *disrupted regulation* group also decreased in touching from 18 to 24 months similar to toddlers, but did not increase in touching from 24 to 36 months, in contrast to the toddlers, but they were touching more at 36 months than older siblings in the *early decrease* group (see Table 2). Older siblings in the *disrupted regulation* group were using significantly more harsh control at 18 months than older siblings in the *early decrease* group but decreased significantly in their harsh control from 18 to 24 months, but not from 24 to 36 months, whereas older siblings in the early decrease group evinced a different pattern, no change from 18 to 24 months, but a significant decline from 24 to 36 months.

A final comparison of multifinality was conducted between the *second‐year peak* and *disrupted regulation* groups because both started similarly at 18 months, but then took very different developmental paths, one increasing then decreasing, and the other decreasing and then increasing. Older siblings in the *second‐year peak* group showed no change in either their touching or use of harsh control from 18 to 24 months, in contrast to the toddlers, but they did show a significant decrease in both from 24 and 36 months at the same time the toddlers were decreasing in their own touching. As Table 2 shows, however, older siblings in the *second‐year peak* group touched the gifts more often at 24 months than older siblings in the *disrupted regulation* group, similar to toddlers, but significantly less at 36 months than older siblings in the *disrupted regulation* group.

#### 3.3.2 Developmental equifinality

The first test of equifinality involved comparing the *late decrease group* with the *second‐year peak* group because the *late decrease* group started high and the *second‐year peak* started low at 18 months, but then both showed a pattern of decline from 24 to 36 months, ending with less touching at 36 months (see Figure 2). Significant changes in the older siblings’ touching in the *late decrease* group mirrored that of the toddlers with a significant decline only from 24 to 36 months, but not from 18 to 24 months. As noted above, older siblings in the *second‐year peak* group did not show a corresponding increase in their touching from 18 to 24 months, but did show a significant decline in touching from 24 to 36 months. Between‐group comparisons revealed no significant differences in older siblings’ touching or harsh control at any time point (see Table 2).

A second test of equifinality compared the *early decrease* and *stable low* groups, and the *late decrease* and *stable low* groups because the low stable group differed at 18 months from the two decreasing groups, but all were low on touching at 36 months, having arrived there by different developmental paths. Older siblings of toddlers in the *stable low* group showed no changes in their touching or harsh control over time, similar to the toddlers; they also touched the gifts significantly less often at 18 months compared to the *early decrease* and *late decrease* groups. Older siblings in the *late decrease* group also used more harsh control than *low stable* older siblings at both 18 and 24 months, and more than older siblings of the *early decrease* group at 18 months, which later declined significantly from 24 to 36 months. However, there were no significant differences in use of harsh control by older siblings in the *low stable* and *early decrease* groups at any time point, even though older siblings in the *early decrease* group were using more harsh control at 18 and 24 months, which later declined significantly from 24 to 36 months (see Table 2).

### 3.4 Parental discipline and toddlers’ behavioral regulation trajectories

In the final analyses, we investigated whether reported PPD toward the two siblings differed over time across the different trajectory groups using RM‐ANCOVAs and whether the differences in older siblings’ behavior across trajectory groups remained when adding PPD as a covariate to the RM‐MANCOVA with older siblings’ touching, harsh and gentle control as dependent variables.

The 3 (time) × 5 (trajectory groups) RM‐ANCOVA (controlling for the age and gender of the older sibling) with parental discipline toward the older sibling as dependent variable showed no significant main effect of time and no significant interaction between time and group (see Table 2). A similar 3 (time) × 5 (trajectory groups) RM‐ANCOVA on punitive discipline toward the toddler, however, did show a significant time x group interaction (see Table 2). Only a linear within‐subject contrast was found (*p* = .015) indicating linear differences over time across groups. Bonferroni‐corrected *post‐hoc* tests showed no significant differences across groups within each time point. As can be seen in Table 2, parents in the *stable low, second‐year peak*, and *early decrease* groups all reported increases in punitive discipline from 18 to 24 months, but only parents in the *second‐year peak* group continued to report an increase in punitive discipline directed toward toddlers from 24 to 36 months. In contrast, parents in the *disrupted regulation* group showed no change in use of punitive discipline from 18 to 24 months but did report using more punitive discipline from 24 to 36 month, in line with the increase in toddler touching from 24 to 36 months in this group.

In an effort to determine if the differences in older siblings’ touching and harsh discipline reported earlier remained once PPD was taken into account, we conducted a final 3 (time) × 5 (trajectory groups) RM‐MANCOVA (older siblings’ touching, harsh and gentle control as dependent variables) controlling for the age and gender of the older sibling and PPD toward both children. All effects reported earlier for older siblings’ touching and control remained significant.

## 4 DISCUSSION

The main goal of this longitudinal investigation was to uncover different developmental trajectories of toddlers’ behavioral regulation from 18 to 36 months in the first study to use a sibling gift‐delay task. We also investigated changes in older siblings’ touching and use of harsh and gentle control during the gift‐delay task that may explain the different developmental trajectories of toddler touching. Finally, we examined if there were differences in the use of PPD across toddler trajectories of behavioral regulation and whether differences in older siblings’ behaviors remained significant once PPD was controlled.

### 4.1 Individual differences in trajectories of toddlers’ behavioral regulation

Five distinct developmental patterns of behavioral regulation were observed. Consistent with developmental trends that children become better able to inhibit responses with age (e.g., Carlson, 2005), two groups of toddlers showed improvement in their behavioral regulation from 18 to 36 months: the *early decrease in touching* (29%) and *late decrease in touching* groups (15%). Similar patterns, reflecting the various patterns of developmental growth in toddlers behavior regulation, were found in a study using a “don't touch” paradigm observing children from 14 to 36 months (Friedman et al., 2011). The third group, representing 31% of toddlers, was the *second‐year peak* group, where toddlers showed a pattern we expected based on prior findings of increases in noncompliant and oppositional behavior in the second year when autonomy seeking and assertiveness emerge (Crockenberg & Litman, 1990; Kuczynski & Kochanska, 1990).

A fourth unexpected trajectory pattern described 11% of toddlers; the *disrupted behavioral regulation* group with toddlers showing a rebound in their touching behavior with an increase from 24 to 36 months after a decline from 18 to 24 months. Because the development of behavioral regulation is a maturational process that transpires over time, and is influenced by socialization experiences, it is possible that these children had not yet consolidated an internalized standard of conduct by 24 months and were still struggling at 36 months to resist temptation. Or perhaps these children by 36 months were capable of understanding right from wrong, but simply refused to comply with their older siblings’ requests, a pattern that has been reported by others studying sibling relationships (Dunn & Kendrick, 1982). Finally, 12% of toddlers showed a stable low pattern of touching, possibly reflecting the early development of behavioral control. This pattern is comparable to results of Dong et al., (2018) using a cleanup task with mothers, who found a group of children who were stably high on committed compliance during the first 3 years.

### 4.2 Older siblings’ touching and use of control during the gift‐delay

A second aim was to consider how the older siblings’ behaviors (touching and control) in the gift‐delay task covaried with the trajectories of toddler touching in an effort to discern how older siblings might be acting as socializing agents, either through modeling, instruction or prohibition. The similarities between the trajectories of toddlers’ touching and changes in their older siblings’ touching over time were quite striking in general, and these results provide some support for the *sibling modeling* hypothesis. Because younger siblings are more inclined to imitate older siblings than vice versa (e.g., Corter, Pepler, & Abramovitch, 1982), toddlers may be mimicking the behaviors of their older siblings during the shared gift‐delay task*.* Both older siblings and toddlers were more likely to touch their own gift than their siblings’ gift. Thus, the similar touching patterns across siblings may reflect the fact that older siblings had difficulties inhibiting their own behavior and touched their gift first with the toddler following suit or that older siblings’ touching was in response to the toddlers’ touching as a means to prevent the toddler from touching both their own and the toddler's gift. Alternatively, the similarities across siblings’ touching might reflect a synchronous set of actions whereby both siblings simultaneously touch their respective toys with no direct influence from the other. Without further information on the sequence of touching (i.e., who touched first), we do not know with certainty which of these possible explanations best represent the findings, and recommend that future research delve further into uncovering these dynamics.

With respect to older siblings’ control strategies, only older siblings’ use of harsh control, not gentle control, differed across the trajectory groups, probably due, in part, to the fact that older siblings used harsh control more than gentle control. In general, older siblings tended to use less harsh control with toddlers across 18 to 36 months, which might be expected with the increased abilities of both children to regulate behavior across this same period of time. In some instances, the harsh control by older siblings corresponded with the extent of, and changes in, toddler's touching (i.e. low stable levels of harsh control in the *low stable* group and stability followed by a decline in harsh control in the *late decrease* group).

However, changes in older siblings’ harsh control did not always match the patterns of toddler touching across time. For instance, older siblings in the *disrupted regulation* group initially showed higher levels of harsh control at 18 months, compared to other groups, and a significant decline in their use of harsh control, in line with the decreased touching of their toddler siblings. But then continued to use less harsh discipline, even though the toddlers increased in their touching. One possible explanation may be that older siblings’ high levels of harsh control at 18 months may have helped suppress toddlers’ touching initially, but if toddlers were reliant on their older siblings’ directives to not touch, the sudden decline in older siblings’ harsh control may have left the toddlers ill‐prepared to regulate their own behavior, which might explain the increase in toddlers’ touching at 36 months (e.g., Cecil et al., 2012). Such initially high levels of harsh control needed to inhibit touching may have interfered with the toddlers’ abilities to internalize a set of standards in the long run. A similar argument has been proposed by Gershoff (2002), pointing to children's initial compliance in response to parental physical punishment but their failure to comply with rules in the absence of such punishment.

### 4.3 Parental Discipline and Toddler Touching

A final aim of this research was to determine if there was any covariation between PPD and the resulting trajectories of toddler touching. Most parents increased significantly in their punitive discipline with toddlers from 18 to 24 months, which may very well reflect normative changes in children's increasing autonomy and parents’ increasing demands for more mature, self‐regulated behavior. These changes in PPD are in line with results of previous longitudinal studies showing increases in parental sternness, negative demeanor, and verbal discipline strategies from 12 to 24 months (Socolar, Savage, & Evans, 2007; Vittrup, Holden, & Buck, 2006). Only parents in the *second‐year peak* group continued to increase their PPD from 24 to 36 months, even though the toddlers were showing significant declines in touching from 24 to 36 months. Perhaps parents applied more punitive disciplinary measures in response to the toddlers’ increasing noncompliance at 24 months, and that the increasing PPD was needed to keep children from increasing in their noncompliance from 24 to 36 months. On the contrary, parents of the *disrupted regulation* group did not change their PPD from 18 to 24 months, but increased their use of PPD significantly from 24 to 36 months congruent to toddlers increased touching. The current analyses do not allow us to infer causal relations so there may be several explanations for these findings that only continuing research can reveal. One possibility is that when parents of these disrupted toddlers refrained from using more PPD from 18 to 24 months when other parents were normatively increasing their use of PPD, they had to compensate by increasing PPD significantly from 24 to 36 months to deal with their toddlers’ disrupted self‐regulation indicated by the increasing pattern of touching while other toddlers were showing a decreasing pattern.

Our final analysis confirmed the potential uniqueness of older siblings’ role in the socialization of their toddlers’ behavioral regulation. This may not be too surprising given that older siblings’ harsh control was unrelated to their parents use of PPD with toddlers. Also, in all cases, older siblings’ use of harsh control decreased from 18 to 36 months, whereas PPD increased during that same period of time. Thus, how older siblings dispense harsh control with their toddler siblings in the sibling gift‐delay appears to be independent of the PPD directed to toddlers at home.

The current findings suggest there are more striking similarities between toddlers’ and older siblings’ touching than between toddlers’ touching and their older siblings’ use of harsh control, providing some support for *sibling modeling* or *sibling synchrony hypothesis*, and more limited support for the *sibling socialization hypothesis,* if we consider the use of control by older siblings during the shared gift‐delay to be a form of socialization. Extant literature provides ample evidence of the direct contributions of older siblings’ behaviors such as prosocial behavior and aggression to younger siblings’ social development (e.g. Pike & Oliver, 2017). Moreover, there was little evidence of social modeling of PPD by older siblings, but this may also be due to differences in methods, because parents’ reports of their parenting behaviors may not always match their actual behaviors.

### 4.4 Limitations

Some limitations and future directions need to be noted. Even though there were clear differences in older siblings’ touching and harsh control that covaried with the trajectories of toddler touching, other factors also may account for these links, such as shared genes and shared environmental exposure (Friedman et al., 2011; Plomin & Daniels, 2011). In addition, differences in toddler temperament, a correlate of early behavioral regulation (Dong et al., 2018; Lehman, Steier, Guidash, & Wanna, 2002), may be related to the diverse developmental trajectories of toddler behavioral regulation. Due to the attrition rate, a common problem of longitudinal studies using observational methods, the sample size was relatively small, which can influence statistical power and increase the likelihood of Type II errors (Banerjee, Chitnis, Jadhav, Bhawalkar, & Chaudhury, 2009). Larger samples and more time points would permit alternative analytic tools that should be explored in future research (e.g., growth mixture models, latent difference scores, measurement burst designs). We also cannot rule out practice and history effects at this time because memory of the events from the prior time point may have affected children's performance in the gift‐delay task at a later time, particularly for the older siblings. But, if that was the case, then we would have expected performance of all children to have improved over time, rather than uncovering the different fluctuating patterns of children's touching over time. Finally, families were predominantly white, college‐educated, and middle‐class and we do not know if similar dynamics would be found between young siblings in families from different ethnic/racial backgrounds, low‐income families or from countries in which sibling caregiving is expected even at young ages (Rabain‐Jamin, Maynard, & Greenfield, 2003). Finally, sibling relationship quality as a moderator of sibling socialization (Pike & Oliver, 2017) may be considered in further research to provide more detailed information of the role of older siblings.

### 4.5 Conclusion

This is the first study to investigate longitudinal variability in the developmental patterns of toddler behavioral regulation during the first three years of life with a focus on sibling socialization. Distinct trajectory patterns were found that underscored the individual differences in toddler behavioral regulation over a critical period (18 to 36 months) for the development of self‐regulation (Kopp, 1982). Both older siblings’ touching and use of harsh control covaried with the trajectories of toddler touching, implicating the role of older siblings as both role models and potential socializers of toddler behavioral regulation. Future research is needed to explore further this uncharted area of toddler development and family influences beyond the parent‐child dyad. Until then, the current study contributes to research on the early development of self‐regulation by underscoring the overlooked role of older siblings as socializers of toddlers’ behavioral regulation.

## Data Availability

The data are not publicly available due to their containing personal data of research participants. The data that support the findings of this study are available from the corresponding author, upon reasonable request.
